# Depletion of the Candida albicans TLO gene family reveals a requirement for alpha TLO genes for wild-type virulence

**DOI:** 10.1099/mic.0.001654

**Published:** 2026-01-16

**Authors:** James O’Connor-Moneley, Theresa Lange, Peter R. Flanagan, Sascha Brunke, Luisa Fischer, Rishabh Sharma, Sumant Puri, Bernhard Hube, Derek J. Sullivan, Gary P. Moran

**Affiliations:** 1 Division of Oral Biosciences, Dublin Dental University Hospital & University of Dublin, Trinity College Dublin, Dublin, Ireland; 2 Department of Microbial Pathogenicity Mechanisms, Leibniz Institute for Natural Product Research and Infection Biology, Hans Knoell Institute (HKI), Jena, Germany; 3 Oral Microbiome Research Laboratory, Kornberg School of Dentistry, Temple University, Philadelphia, PA, USA; 4 Institute of Microbiology, Friedrich Schiller University, Jena, Germany; 5 Cluster of Excellence Balance of the Microverse, Jena, Germany

**Keywords:** *Candida albicans*, gene expression, *TLO* genes, virulence

## Abstract

*Candida albicans* uniquely possesses an expanded family of genes (the *TLO* gene family) that encodes 10–15 paralogues of the Med2 component of the transcriptional regulator Mediator. Previous studies have shown that *TLO* null mutants are unable to form hyphae and are hypersensitive to environmental stress. However, the reason for the *TLO* gene expansion remains unclear, and the current study aimed to determine if reduction in the *TLO* family copy number affected virulence. In order to investigate this, we used CRISPR-Cas9 mutagenesis to generate two *TLO*-depleted mutants: one mutant retaining only *TLO*β*2* (CaTLO2) and the second mutant containing only *TLO*γ*5* (CaTLO5). Both *TLO*-depleted mutants exhibited increased filamentous growth, increased susceptibility to specific stresses and reduced virulence in a murine model of oropharyngeal candidiasis (OPC). *In vitro*, the CaTLO5 mutant also exhibited impaired hyphal escape from macrophages and reduced hyphal invasion of oral keratinocytes. We then investigated if complementation with *TLO*α*1*, a gene previously shown to restore wild-type growth in a Δ*tlo* null mutant, could restore virulence. *In vitro* infection models showed that *TLO*α*1* could restore true hypha formation, epithelial invasion and hyphal escape from macrophages in the CaTLO5 background. The murine OPC model showed that *TLO*α*1* could restore wild-type virulence in both CaTLO2 and CaTLO5 strains, suggesting an essential role for α-*TLO* in oral mucosal infection. Together, these findings highlight the functional specialization between the α, β and γ *TLO* gene groups and establish α-*TLO* as a major regulator of virulence in *C. albicans*.

## Data Summary

All raw data are available upon reasonable request. All RNA-seq data are available from the NCBI Sequence Read Archive, BioProject number PRJNA1266650 under the accession numbers SRX28893235, SRX28893236, SRX28893237, SRX28893238, SRX28893239, SRX28893240, SRX28893241, SRX28893242, SRX28893243, SRX28893244, SRX28893245, SRX28893246, SRX28893247, SRX28893248 and SRX28893249.

## Introduction

*Candida albicans* is an opportunistic fungal pathogen that colonizes the mucosal surfaces of healthy individuals but can also cause superficial or even severe systemic infections, particularly in immunocompromised hosts [1]. Its ability to shift between commensal and pathogenic lifestyles is underpinned by remarkable phenotypic plasticity, allowing rapid adaptation to diverse host environments [1,2]. Critical to this adaptability is the fungus’s capacity to transition between yeast and filamentous growth morphologies, form biofilms, tolerate oxidative and nutritional stress and modulate immune responses [1,3, 4]. These virulence traits are tightly regulated by complex gene networks, with the Mediator transcriptional coactivator complex playing a central role in integrating environmental cues and controlling downstream gene expression [5,9].

A notable feature of the *C. albicans* genome is the expansion of the *TLO* (Telomere-associated) gene family, which encodes homologues of *MED2*, a subunit of the Mediator tail module [8]. While *Saccharomyces cerevisiae* and most other eukaryotic species encode a single *MED2* gene, *C. albicans* strains possess 10–15 *TLO* paralogues, nearly all located near subtelomeric regions [10,11]. These genes are grouped into three architectural groups (previously referred to as clades) – α, β and γ – based on their sequence variation and structural motifs [12], and genomic analyses have shown that the *TLO* copy number can vary between strains due to telomeric recombination [10,12, 13]. Strain SC5314 possesses 14 *TLO* genes including 6 α-*TLO* genes, 1 β-*TLO* gene and 7 γ-*TLO* genes. The evolutionary drivers and functional significance of this gene expansion have been the focus of increasing investigation [12,15]. Functional studies revealed that *TLO* genes are required for numerous virulence-related phenotypes, including filamentation, stress resistance and host interaction [10,14,19].

Our previous studies have shown that a *Δtlo* null mutant is defective in transcriptional responses, does not form true hyphae in serum and is hypersusceptible to environmental stress [15]. These phenotypes could be complemented by *TLO*α*1* (as a representative of the α-subgroup) and *TLO2* (as the sole β-subgroup member), with the *TLO*β*2* gene also promoting filamentous growth. However, the γ genes (represented by *TLO*γ*5* and *TLO*γ*11*) only weakly complemented the Δ*tlo* mutant phenotypes. Our previous studies did not directly address the requirement of *TLO* for virulence, largely due to the extremely compromised growth of the *Δtlo* mutant which rendered these analyses redundant.

However, the current study aimed to investigate a more pertinent question, i.e. whether an expanded number of *TLO* genes was required for wild-type virulence. To investigate the role of the *TLO* expansion and diversification of the family into α, β and γ subgroups, we generated strains in which the *TLO* gene family was systematically depleted to retain only a single homologue. Despite the high level of sequence homology, we were able to design two different gRNA molecules that facilitated the deletion of almost all *TLO* genes with the exception of either *TLO*β*2* or *TLO*γ*5*. The *TLO*β*2* and *TLO*γ*5* paralogues have previously been shown *in vitro* to be functionally distinct, with *TLO*γ*5* only weakly complementing *Δtlo* mutant phenotypes and *TLO*β*2* restoring a phenotype similar to wild-type but with excess filamentous growth. Construction of these mutants allowed us to investigate if retention of a single *TLO* β or γ gene was sufficient for virulence. Furthermore, this strategy allowed us to further examine whether *TLO* expansion via reintegration of *TLO*α*1*, an α-*TLO* gene previously shown to regulate morphogenesis and stress responses, was sufficient to enhance virulence-associated phenotypes.

Together, these results provide new insights into the functional specialization of the *TLO* α, β and γ genes. They confirm that the different subgroups possess distinct roles in coordinating virulence in *C. albicans*. More broadly, our work illustrates how gene family expansion can support regulatory plasticity and pathogenic fitness in fungal pathogens through additive mechanisms.

## Methods

### Strains and growth conditions

*C. albicans* strains in this work (Table S1, available in the online Supplementary Material) were cultured in Yeast Extract Peptone Dextrose (YEPD) medium (liquid or solid) at 37 °C. To quantify fungal cell morphology in liquid cultures, three fields (×20 magnification) per strain were assessed. All cells were counted in each field (containing at least 100 cells) to enumerate the proportions of yeasts, hyphae and pseudohyphae. Experiments were carried out on at least three occasions. To determine growth rates in liquid YEPD, cells were washed in sterile water, and a suspension was prepared in YEPD at OD_600_ 0.1, and 120 µl volumes were added to 96-well flat-bottom plates. Plates were incubated in a FLUOstar Omega Microplate reader for 24 h at 200 r.p.m., 37 °C, and the OD_600_ values were determined hourly. Doubling times were calculated from the exponential phase using GraphPrism v10 (San Diego, CA, USA). Hyphal growth was induced by inoculating cells grown overnight in YEPD at 30 °C in fresh YEPD with 10% (v/v) FBS at an OD_600_ of 0.2. Cultures were incubated at 37 °C in either a static or a shaking incubator, and hyphal growth was monitored using a Zeiss AX10 stereomicroscope. For calcofluor white (CFW) staining, 5 µl of cell culture was mixed with 5 µl of CFW stain (Sigma-Aldrich). Spot plate assays to assess susceptibility to oxidative stress and cell wall-damaging agents and biofilm formation assessment in Spider medium using crystal violet staining were all performed as described by Flanagan *et al*. [16]. Fluconazole tolerance, including measurement of Fraction of Growth (FoG) inside the zone of inhibition and the Radius (RAD) of clearance, was determined as previously described [17].

### CRISPR-Cas9 mutagenesis and complementation

CRISPR-Cas9 mutagenesis was performed using the pV1200 ‘solo’ vector system described by Vyas *et al*. [20]. Manual inspection of *TLO* gene alignments was used to identify prospective guide RNA (gRNA) molecules that targeted all *TLO* genes with single exceptions. Due to the high level of sequence homology within the *TLO* gene family, the choice of paralogues to retain was limited due to the level of sequence conservation. However, our analysis resulted in the identification of Guide 1 (5′-AATGATGCAGAGTGGTGTCT) which targeted all *TLO* genes apart from *TLO*β*2* and Guide 2 (5′-ATTGAAAGTCAAAGAAGAAG) that targeted all genes apart from *TLO*γ*5*. These gRNAs were cloned in pV1200 according to the methods of Vyas *et al*. [20].

Transformation was carried out with pV1200 and the *TLO* deletion repair template described by Fletcher *et al*. [15] by electroporation as described by Moran *et al*. [21], and NAT marker recycling was carried out in BSA medium as described by Staab *et al*. [22]. Screening for *TLO* deletion mutants was carried out as described by Fletcher *et al*. [15] by using the ‘pan-*TLO*’ primer in conjunction with locus-specific primers for each *TLO* gene, which yielded a single truncated PCR product in strains with a homozygous deletion in the specific *TLO* gene [15]. The *TLO*-depleted mutants were complemented with the *TLO*α*1* ORF cloned in the pNIM1 vector, as described by Fletcher *et al*. [15].

### RNA-seq analysis and quantitative RT-PCR

Overnight cultures were grown from single colonies in 4 ml YEPD at 37 °C with 200 r.p.m. shaking, and fresh YEPD was inoculated from the overnight cultures to an OD_600_ of 0.1 and incubated at 37 °C with 200 r.p.m. shaking until an OD_600_ of 0.8 was reached. RNA was extracted from strains using the RNeasy extraction kit (QIAGEN) per the manufacturer’s instructions. mRNA sequencing was performed in biological triplicate experiments with strand-specific libraries and sequenced on the Illumina NovaSeq 6000 Sequencing System using paired-end 150 bp reads. Each experiment generated a minimum of >20 million read pairs per sample with Q30 score ≥85%. Raw reads were aligned using HISAT2 [23] to the *C. albicans* SC5314 Assembly 21 genome [downloaded from the *Candida* Genome Database (CGD)], and reads were quantified and normalized using DeSeq2 [24] . Statistical analysis of differential expression was carried out with three biological replicates with post-hoc Benjamini–Hochberg testing performed by default (FDR q<0.05). Further analysis on lists of differentially expressed genes was performed *via* GO analysis on the CGD database and Gene Set Enrichment Analysis (GSEA) [15,18]. RNA sequencing data are available from NCBI, accession number PRJNA1266650.

The expression of the *TLO* mRNA was determined by qRT-PCR. cDNA was generated from RNA extracted using the RNeasy kit (Qiagen) from strains grown in YEPD medium at 37 °C with 200 r.p.m. shaking until an OD_600_ of 0.8 was reached. Specific qRT-PCR primers for *ACT1* were described by Fletcher *et al*. [15]. For quantification of total *TLO* expression, we designed a set of primers that aligned to all *TLO* genes (AAK_27 : 5′-ACTAGCCCCAACAACGAAC and AAK-28: 5′-TTTCAACCATAACGCCGAGAC). All qRT-PCR reactions were carried out in biological triplicate using an Applied Biosystem 7500 Fast Real-Time PCR System, with the expression of *ACT1* as an endogenous control. Differential expression was calculated as described by Schmittgen and Livak [25].

### Culture of oral epithelial cells

The human oral epithelial cells (TR146, European Collection of Authenticated Cell Cultures ECACC #10032305) were routinely cultivated at 37 °C and 5% CO_2_ in Dulbecco’s modified Eagle’s medium/Nutrient Mixture F-12 (DMEM/F12, Life Technologies) supplemented with 10% FBS (Bio and Sell) for no longer than 15 passages. For damage assays, TR146 cells were seeded at a total concentration of 2×10^4^ cells/well in a 96-well plate. For invasion/adhesion/filamentation assays, 1×10^5^ TR146 cells were seeded on a coverslip in a 24-well plate. Confluent TR146 cells were washed once, and subsequent experiments were carried out in serum-free medium.

### Monocyte isolation from buffy coats and macrophage differentiation

Human peripheral blood was collected from healthy volunteers with ethics approval and after obtaining written informed consent. This study was conducted according to the principles expressed in the Declaration of Helsinki. The blood donation protocol and use of blood for this study were approved by the institutional ethics committee of the University Hospital Jena (permission number 2207–01/08). Preparation of human monocyte-derived macrophages (hMDMs) was performed as described before [26] based on selection of monocytes by magnetic automated cell sorting of CD14-positive monocytes and a differentiation period of 7 days. Adherent hMDMs were detached with 50 mM EDTA in PBS and seeded in 96-well plates (4×10^4^ hMDMs/well) or in 24-well plates on coverslips (2×10^5^ hMDMs/well). Seeding was performed in RPMI 1640 (Gibco, Thermo Fisher Scientific) supplemented with 10% FBS and 50 ng ml^−1^ M-CSF (ImmunoTools). Incubation of hMDMs was always performed at 37 °C and 5% CO_2_ overnight. Before infection with *C. albicans*, the previous medium was removed, hMDMs were washed once with PBS, and RPMI without FBS was added.

### Human cell damage assay

Human cells were seeded in a 96-well plate as described above, infected with *C. albicans* (MOI of 1 for epithelial cells and MOI of 5 for hMDMs) and incubated for 24 or 48 h. Release of the cytoplasmic enzyme lactate dehydrogenase (LDH) was measured as a marker for necrotic epithelial damage [27] using a Cytotoxicity Detection Kit (Roche) according to the manufacturer’s instructions. The LDH release was expressed as % of high (full lysis) control (maximum LDH release induced by the addition of 0.5% Triton X-100 to uninfected epithelial cells for 10 min) unless otherwise stated.

### Survival in macrophages

*C. albicans* was grown overnight in liquid YPD medium at 180 r.p.m. at 30 °C. Yeast cells were then harvested by centrifugation (20,000 ***g***, 1 min) and washed twice with PBS, and the yeast cell number was adjusted. hMDMs were infected with an MOI of 5. To determine intracellular fungal survival 3 h post-infection, the supernatant was removed, and macrophages were lysed by adding 200 µl ddH_2_O, scraping the well and pipetting up and down to break the cells. This was repeated with each well five times. The supernatant and lysate were each appropriately diluted with PBS and plated on YPD plates. The plates were incubated at 30 °C, and colony-forming units (CFUs) were determined after 2 days to assess fungal survival.

### Quantification of hypha formation, invading/escaping hyphae and hyphal length

Human cells were infected in a 24-well plate with *C. albicans* cells at an MOI of 1 for epithelial cells and MOI of 5 for hMDMs as described above and incubated for 3 h. Non-adherent *C. albicans* cells were removed by rinsing with PBS, and samples were fixed with Roti^®^-Histofix 4% (Roth). Extracellular, non-invasive fungal components were stained with AlexaFluor647 conjugate of succinylated concanavalin A (ConA; Invitrogen). After rinsing with PBS, host cells were permeabilized with 0.5% Triton X-100 for 10 min. Next, the entire fungal cells (invasive and noninvasive) were stained with CFW (Sigma-Aldrich) and visualized by fluorescence microscopy (Leica DM5500B, Leica DFC360 FX). The total hyphal length was measured using the Leica LAS AF software (Leica Microsystems CMS GmbH), as well as the percentage of filaments and invasive/escaping hyphae (only CFW-stained), counted from at least 100 hyphae per strain for each biological replicate.

For high-quality images, human cells were infected in a 24-well plate with *C. albicans* cells at an MOI of 1 for epithelial cells and MOI of 5 for hMDMs as described above and incubated for 3 h. Non-adherent *Candida* cells were removed by rinsing with PBS, and samples were fixed with Roti^®^-Histofix 4% (Roth). The host cells were permeabilized with 0.5% Triton X-100 for 10 min. Next, the entire fungal cells were stained with AlexaFluor647 conjugate of succinylated ConA. After rinsing with PBS, the host and fungal nuclei were stained with 5 µg ml^−1^ DAPI (D9542, Sigma-Aldrich) per well. The coverslips were mounted on microscopy slides, and imaging was performed on the Zeiss Axio Observer.

### Murine oropharyngeal candidiasis model

An immunosuppressed murine model of oropharyngeal candidiasis (OPC), previously described by Sharma *et al*. [28], was used. Briefly, female C57BL/6 mice (4 to 6 weeks old) were immunosuppressed by subcutaneous injection of 225 mg kg^−1^ of cortisone acetate (C3130; Sigma) on days −1, 1 and 3. Mice were provided with 2% dextrose in their drinking water from days −1 to 5. On the day of infection (0 day), mice were anaesthetized with ketamine:xylazine (10 : 1) and sublingually infected with 5×10^6^ cells ml^−1^ of *C. albicans* strains for 45 min. On day 5 post-infection, mice were euthanized, and tongue tissue was collected, homogenized in PBS, serially diluted and plated on YEPD agar supplemented with streptomycin/penicillin (SV30010; HyClone). Plates were incubated at 30 °C for 48 h. Fungal burden in tongue tissue was quantified as mean log10 values of c.f.u. All animal procedures were conducted in accordance with ethical guidelines and approved by the Temple University Institutional Animal Care and Use Committee (IACUC protocol #5079).

### Statistics and reproducibility

Experiments were performed in at least biological triplicates (*n*=3) with three independent experiments. For experiments using primary macrophages at least four different donors were used. Data were analysed using GraphPad Prism 10.0.1 (GraphPad Software, La Jolla, CA, USA). Values are presented as mean±sd. Statistical tests are indicated in each figure legend. Statistical significance is indicated in the figures as follows: *, *P*≤0.05; **, *P*≤0.01; ***, *P*≤0.001; ****, *P*≤0.0001.

## Results

### Phenotypic characterization of *TLO*-depleted *C. albicans* mutants

In order to investigate the impact of depleting the copy number of the *TLO* gene family, we used CRISPR-Cas9 mutagenesis to generate a set of *C. albicans* strains where the number of functional *TLO* genes was reduced from 14 to a single intact gene (i.e. *TLO*β*2* and *TLO*γ*5*). We designed two unique gRNAs that targeted all *TLO* genes in SC5314 with the exception of a single member: gRNA1 was used to introduce a deletion in all *TLO* genes apart from the *TLO*β*2* gene to generate strain CaTLO2, and gRNA2 was used to introduce a deletion in all *TLO* genes apart from *TLO*γ*5*, generating strain CaTLO5 (Table S1). Genotypes were determined by locus-specific PCR (Fig. S1). Next, we reintroduced the *TLO*α*1* gene in both CaTLO2 and CaTLO5 under the control of the doxycycline-inducible *pTET* promoter using the pNIM1 plasmid, thereby generating strains that express two *TLO* genes from different groups, which we termed CaTLO2+1 and CaTLO5+1 (Table S1). We have previously shown that the leaky *pTET* promoter yields sufficient expression of *TLO*α*1* mRNA and protein to support wild-type growth [15] and all experiments were carried out without doxycycline, unless indicated. As a control, we used the parental strain SC5314 or a derivative of SC5314, CapV1200, that harbours the remnants of the recycled Cas9 plasmid at the *ENO1* locus (Table S1).

**Fig. 1.**
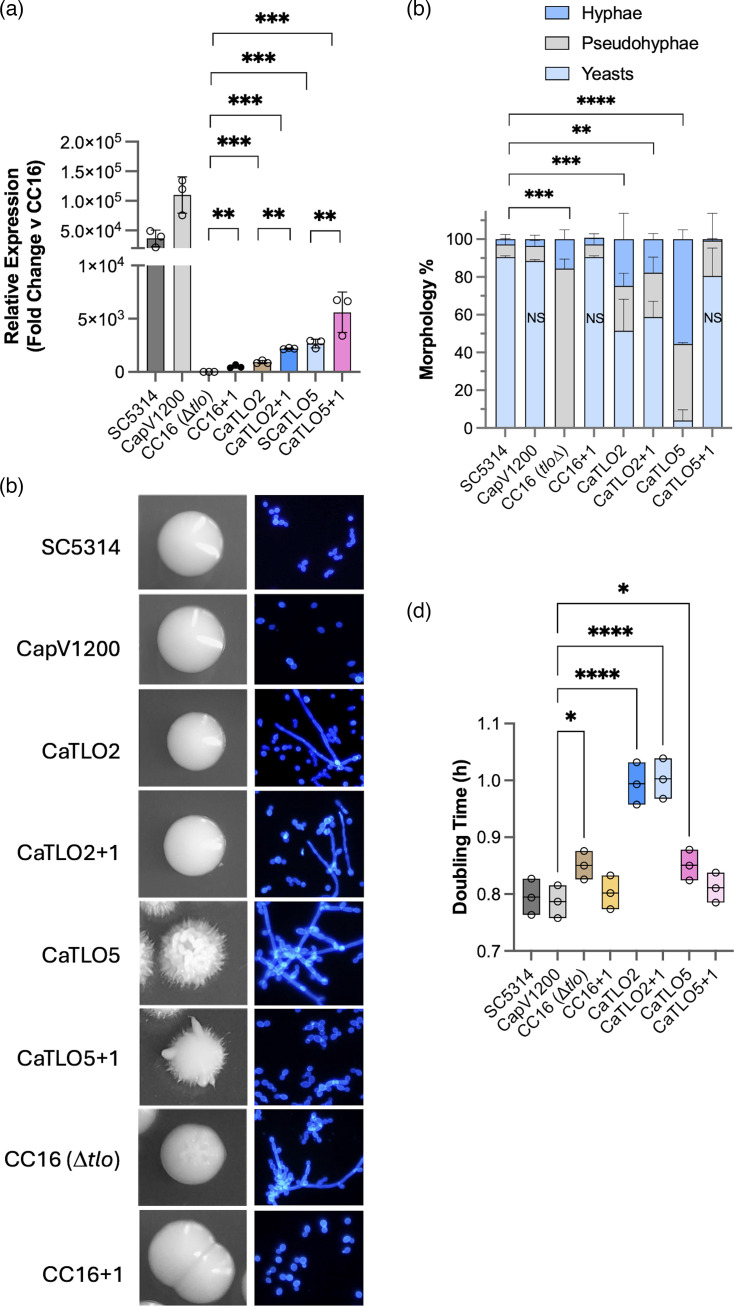
*TLO* expression levels and morphological characterization of *TLO*-depleted and complemented *C. albicans* mutants. (**a**) Quantitative RT-PCR analysis of total *TLO* expression in the indicated strains grown to exponential phase in YEPD medium at 37 °C. Gene expression was normalized to *ACT1* and expressed as fold-change relative to CC16. Data represent mean±sd from three independent biological replicates and significant differences from SC5314 identified using ANOVA with post-hoc Benjamini–Hochberg testing. (**b**) Colony and cellular morphology of each strain grown on YEPD agar at 37 °C for 48 h, along with CFW-stained images of cell morphology. (**c**) Proportion of yeasts, hyphae and pseudohyphae in mid-exponential YEPD cultures at 37 °C, estimated by counting 100 cells in at least 3 fields (see methods). Data were analysed using 2-way ANOVA comparing strain and morphology (yeasts, hyphae or pseudohyphae) with Dunnett’s multiple comparison test. Asterisks indicate the significance of the differences in numbers of yeast cells detected in each strain compared to SC5314. (**d**) Doubling times of each strain, calculated from the exponential phase of growth curves. Box plots show mean, maximum and minimum values from three independent biological replicates analysed using ANOVA. Statistical significance is indicated by asterisks: *P<0.05, **P<0.01, ***P<0.001 and ****P<0.0001.

Quantifying total *TLO* gene expression in CaTLO2 and CaTLO5 using qRT-PCR confirmed significantly reduced *TLO* mRNA levels associated with *TLO* depletion and a subsequent increase in *TLO* expression following the addition of *TLOα1* (Fig. 1a). Morphologically, the colonies formed by CaTLO2 on YEPD agar at 37 °C exhibited the same smooth appearance as colonies formed by both parental control strains (Figs 1b and S2). However, when cells from CaTLO2 colonies were examined under the microscope, they were found to contain a mixture of yeast cells and hyphal cells (Fig. 1b). Integration of *TLO*α*1* to generate CaTLO2+1 did not appear to have any impact on colony or cellular morphology (Fig. 1b). In contrast, CaTLO5 grew as wrinkled colonies with hyphal fringes. This is likely due to the fact that CaTLO5 colonies, in contrast to CaTLO2 colonies, consisted almost entirely of filamentous cells, even at 30 °C (Figs 1b and S2). Reintegration of *TLO*α*1* in CaTLO5 partially restored smooth colony morphology and yeast cell growth, but colonies still maintained the hyphal fringe (Fig. 1b). Complementation of a *Δtlo* strain with *TLO*α*1* restored a wild-type colony and cellular morphology, emphasizing the blended nature of the phenotypes in the CaTLO2+1 and CaTLO5+1 strains. An accurate quantification of the proportions of yeasts, hyphae and pseudohyphae was carried out in liquid YEPD cultures (Fig. 1c). This analysis showed that the CaTLO2, CaTLO2+1 and CaTLO5 strains all exhibited significantly lower abundances of yeasts relative to the parent SC5314, whereas the *TLO*α*1*-complemented strain CaTLO5+1 was not significantly different to SC5314 in terms of proportions of cell morphologies (Fig. 1c).

Comparison of growth curves in liquid YEPD at 37 °C showed that the *TLO*-depleted strains grew more slowly than the control strains, which may partly be linked to filamentous growth (Fig. S3). CaTLO2 exhibited the most significant increase in doubling time relative to SC5314, whereas CaTLO5 exhibited a smaller increase and was comparable to the Δ*tlo* mutant (Fig. 1d). Reintegration of *TLO*α*1* restored the doubling time in CaTLO5 to a level similar to SC5314, whereas growth of CaTLO2 was not significantly affected by the reintroduction (Fig. 1d).

In YEPD supplemented with 10% serum, SC5314, CaTLO2 and CaTLO2+1 all formed abundant true hyphae within 2 h; however, the CaTLO5 strain remained locked in a pseudohyphal morphology (Fig. S4). In CaTLO5+1, the introduction of *TLO*α*1* was able to restore hyphal growth to a level similar to the isogenic control strain CapV1200 (Fig. S4). To determine whether *TLO* depletion influences biofilm development, static biofilm assays were carried out. The Δ*tlo* mutant, as previously reported [15], formed significantly less biofilm than SC5314. The *TLO*-depleted strains CaTLO2 and CaTLO5 also exhibited significantly reduced biofilm formation compared to SC5314 (Fig. S5). Reintegration of *TLO*α*1* into these strains restored biofilm formation to wild-type levels.

**Fig. 2.**
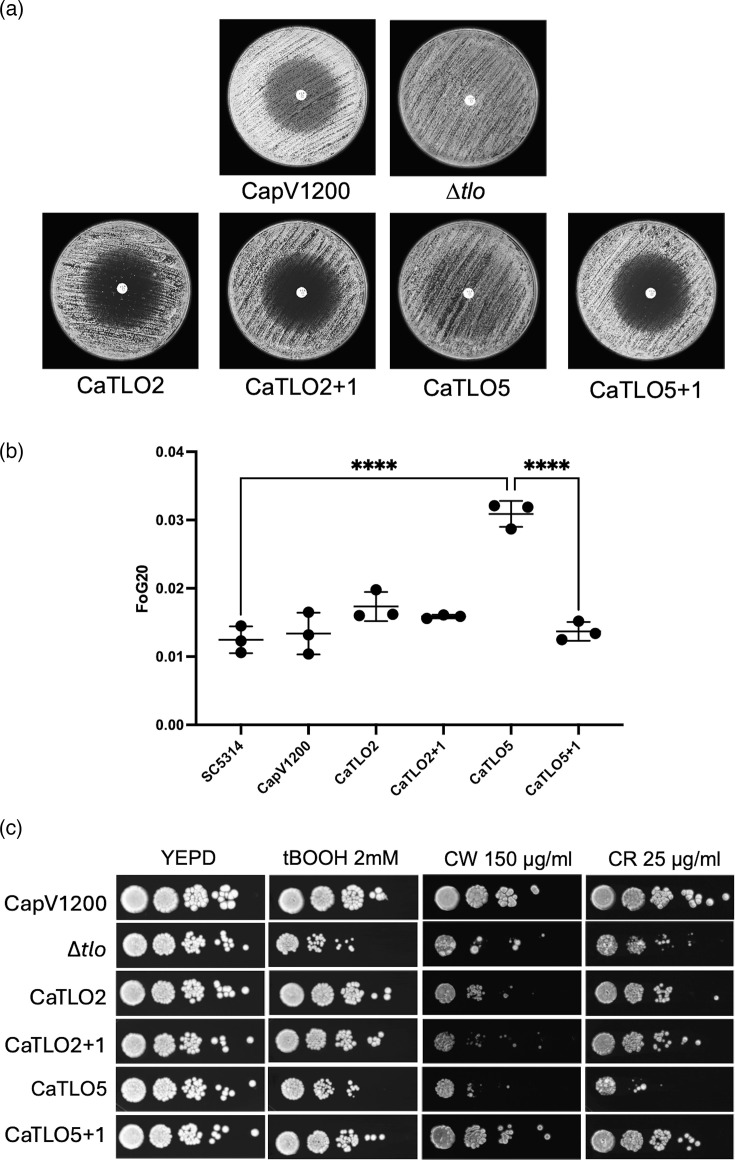
Fluconazole tolerance and stress sensitivity of *TLO*-depleted and complemented *C. albicans* strains. (**a**) Representative fluconazole (FLC) disc diffusion assays performed on RPMI-1640 agar plates following 48 h of incubation at 37 °C. (**b**) Quantification of FoG within the zone of inhibition (FoG20) for each strain, calculated from disc diffusion plates shown in (**a**). Data represent mean±sd from three independent biological replicates. Statistical significance was determined by one-way ANOVA with Tukey’s multiple comparisons test; ****P<0.0001. (**c**) Spot plate assays assessing oxidative and cell wall stress tolerance. Strains were spotted in serial dilutions onto YEPD (control), YEPD supplemented with 2 mM tBOOH (oxidative stress), 150 µg ml^−1^ CFW or 25 µg ml^−1^ CR. Plates were incubated at 37 °C for 48 h.

Given the clinical relevance of antifungal tolerance and resistance, we assessed sensitivity to fluconazole using fluconazole disc diffusion assays (Fig. 2a). Only CaTLO5 exhibited increased fluconazole tolerance compared to SC5314, a phenotype previously observed in the ∆*tlo* null mutant [17]. Reintroducing *TLO*α*1* restored SC5314-like susceptibility in CaTLO5 (Fig. 2a, b). Spot plate assays also revealed changes in oxidative and cell wall stress tolerance across different *TLO*-depleted mutants and *TLO*α*1* reintegrants (Fig. 2c). CaTLO5 displayed increased sensitivity to oxidative stress (2 mM tBOOH) and cell wall stressors [CFW and Congo Red (CR)] in spot plate assays, which was confirmed with MIC assays (Fig. S6). Sensitivity to tBOOH and the cell wall stressors was reversed upon *TLO*α*1* reintegration (Fig. 2c). Interestingly, CaTLO2 exhibited an increased sensitivity to cell wall stress agents, which was not fully rescued by *TLO*α*1*, suggesting that there may be distinct regulatory pathways involving *TLO*β*2* (Fig. 2c).

### *TLO*α*1* and *TLO*β*2* expression interplay governs hyphal morphogenesis

**Fig. 3.**
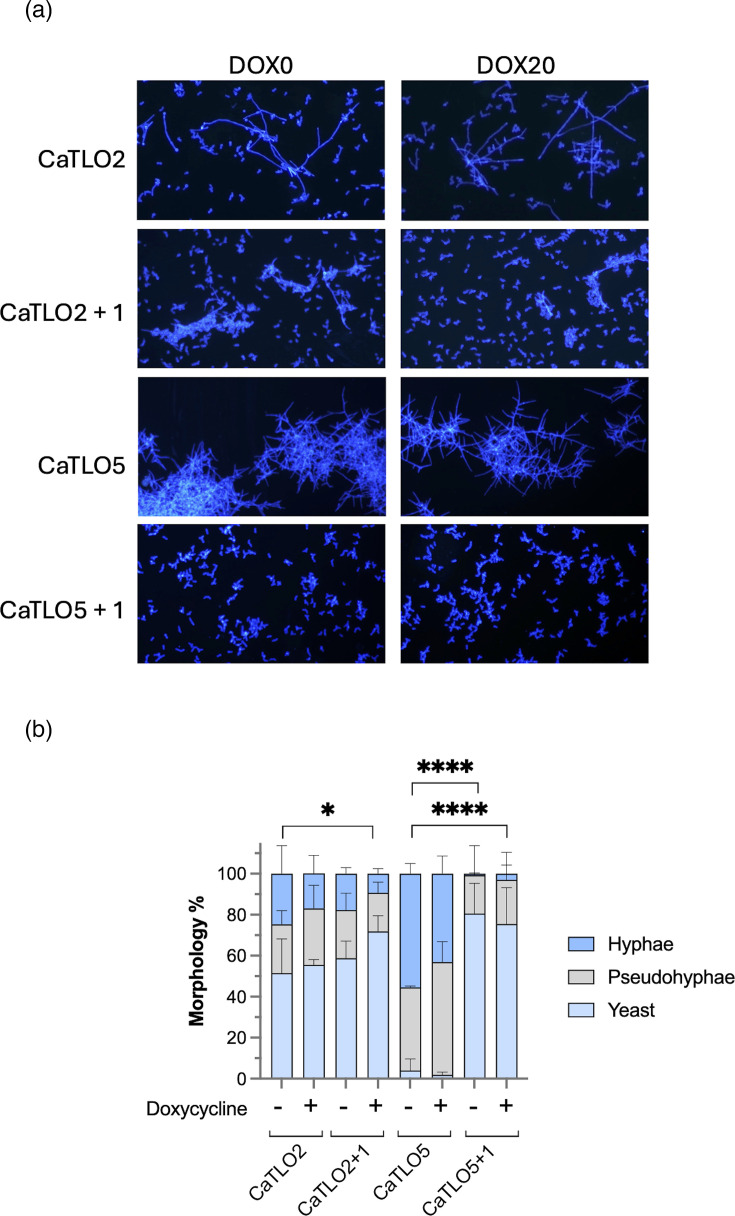
Effect of doxycycline-induced *TLO*α*1* overexpression on hyphal morphology in *TLO*-depleted *C. albicans* mutants. (**a**) Representative CFW-stained micrographs showing morphology of *TLO*-depleted mutants (CaTLO2, CaTLO5) and *TLO*α*1* reintegrants (CaTLO2+1, CaTLO5+1) cultured in YEPD medium at 37 °C to mid-exponential phase in the presence or absence of doxycycline (20 µg ml^−1^). (**b**) Proportion of yeasts, hyphae and pseudohyphae in cultures from (**a**), estimated by counting 100 cells in at least 3 fields. Asterisks represent significant differences in the proportions of yeasts compared to CaTLO2 or CaTLO5, as indicated, determined using 2-way ANOVA with Dunnett’s multiple comparison test.

We next investigated the effect of *TLO*α*1* expression level on the cellular morphology of reintegrant *C. albicans* strains. Although *TLO*α*1* was able to restore almost wild-type morphology in the CaTLO5 background, the expression of *TLO*α*1* in the *TLO*β*2*-expressing CaTLO2 mutant did not restore wild-type growth rates or wild-type cellular morphologies. We hypothesized that in the presence of Tloβ2, higher levels of Tloα1 expression may be required to observe α*-TLO* gene phenotypes. To this end, we tested whether increased doxycycline-induced *TLO*α*1* expression levels could promote a wild-type-like yeast cell phenotype associated with α*-TLO* gene expression. For this analysis, the *TLO*-depleted strains and their corresponding *TLO*α*1* reintegrants were cultured in YEPD media to mid-exponential phase in the presence or absence of doxycycline and their morphology examined (Fig. 3a). The results demonstrated that doxycycline induction of *TLO*α*1* expression partially inhibited the hyphal phenotype of the CaTLO2 mutant, resulting in a significant increase in the proportion of yeast cells in cultures (Fig. 3b). In contrast, doxycycline exposure had no observable effect on the morphology of other strains; CaTLO5 maintained its hyphal phenotype in the presence of doxycycline, and CaTLO5+1 consistently lacked hyphae due to the restoration of yeast growth following *TLO*α*1* reintegration. These findings suggest that high levels of Tloα expression (represented here by *TLO*α*1*) are required to maintain yeast-phase morphology in the presence of Tloβ2, the sole gene detected in the CaTLO2 background.

### Transcriptomic analysis of *TLO-*depleted mutants and *TLO*α*1*-complemented strains

**Fig. 4.**
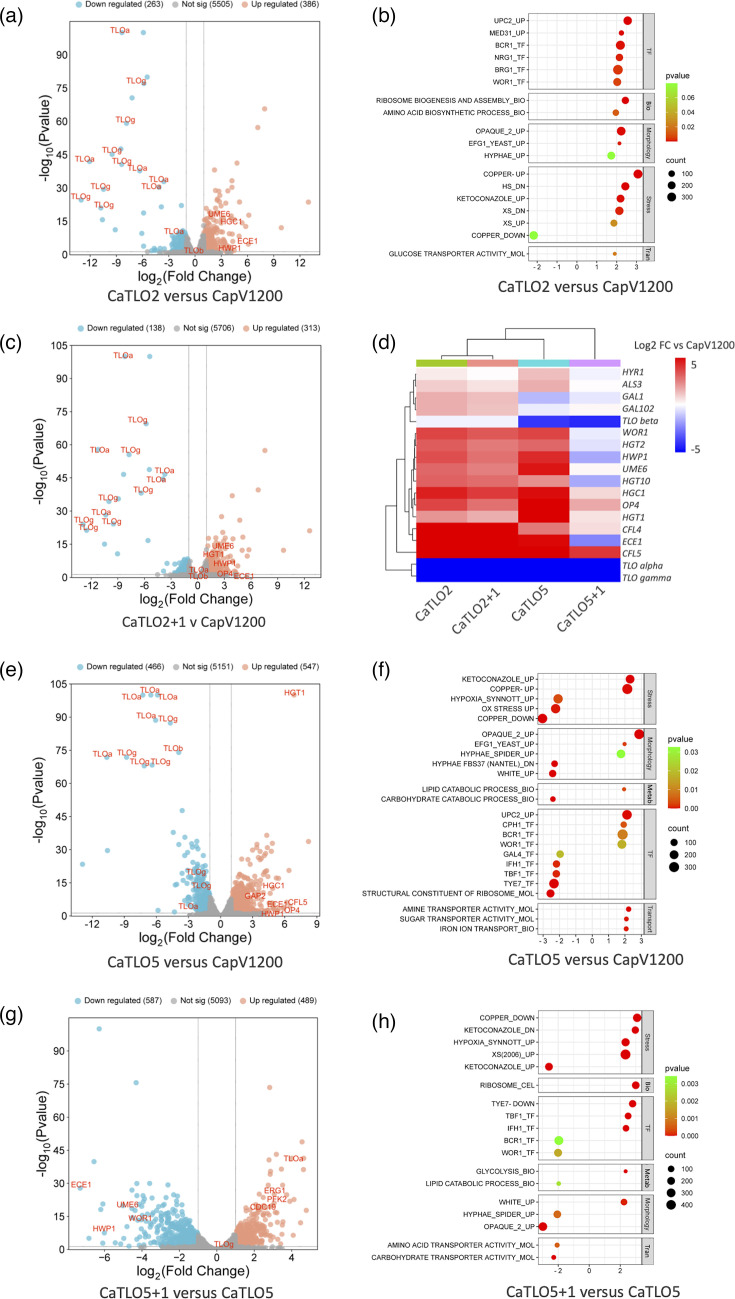
Transcriptomic analysis of *TLO*-depleted and complemented *C. albicans* mutants. Volcano plots show the position of genes highlighted in the text and significantly expressed α, β and γ *TLO* paralogues (FDR *P*<0.1). (**a**) Volcano plot showing differentially expressed genes in CaTLO2 compared to the CapV1200 wild-type strain. (**b**) GSEA of CaTLO2 versus CapV1200. (**c**) Volcano plot of differentially expressed genes in CaTLO2+1 compared to CapV1200. (**d**) Heatmap showing expression of selected genes in *TLO* mutants relative to CapV1200. (**e**) Volcano plot comparing CaTLO5 and CapV1200. (**f**) GSEA of CaTLO5 versus CapV1200. (**g**) Volcano plot comparing CaTLO5+1 and CaTLO5. Only two significantly expressed (FDR *P*<0.1) *TLO* paralogues were identified. (**h**) GSEA of CaTLO5+1 versus CaTLO5. GSEA gene categories represented include ChIP targets of specific transcription factors (TF), Biosynthesis genes (Bio), Morphology-related genes, Stress Responses, Metabolism (Metab) and Transport (Tran).

We conducted transcriptomic profiling of the *TLO*-depleted strains and their respective *TLO*α*1* reintegrants to investigate the molecular basis of the phenotypes we observed. Although short-read sequencing is unable to effectively discriminate between the highly similar *TLO* paralogues, we could detect gross changes in α- or γ-group *TLO* gene expression in the respective mutants. Comparison of the CaTLO2 mutant to the isogenic control strain CapV1200 showed a general reduction in expression of mRNAs homologous to *TLO*α and *TLO*γ genes in CaTLO2 (Fig. 4a). These experiments also reflected the presence of hyphae in CaTLO2 cultures (grown in the absence of serum) as there was an increased expression of the transcription factor gene *UME6*, which is involved in regulating filamentation [29]; *HGC1*, which is required for hyphal growth, biofilm formation and virulence in mice [30]; *ECE1*, which encodes candidalysin, a major virulence factor associated with the hyphal invasion of host cells [31]; and *HWP1*, which is also associated with the hyphal cell wall [22] (Fig. 4a, d). GSEA further demonstrated changes of various biological processes in the CaTLO2 mutant compared to the wild-type. These included increased expression of genes related to hyphal morphology, stress responses and metabolic processes in the CaTLO2 mutant compared to the isogenic control strain, CapV1200 (Fig. 4b).

Comparison of the gene expression profiles of the CaTLO2 mutant and the CaTLO2+1 reintegrant demonstrated that reintegration of *TLOα1* in this background had no significant effect on the transcriptional profile of the CaTLO2 mutant (Fig. S7). Accordingly, when comparing the transcriptional profile of the CaTLO2+1 reintegrant with the isogenic control CapV1200, many of the key genes that were upregulated in the CaTLO2 mutant were also upregulated in the CaTLO2+1 reintegrant, including *UME6*, *HWP1* and *ECE1* (Fig. 4d). Equivalent expression of *TLO*α gene transcripts was detected in the CaTLO2+1 reintegrant and the CapV1200 strain, suggesting successful reintegration and expression of *TLO*α*1* in the CaTLO2 background (Fig. 4c).

Comparison of the transcriptomic profile of the CaTLO5 mutant to CapV1200 revealed that similar to CaTLO2, the CaTLO5 mutant exhibited upregulation of *HGC1*, *ECE1* and *HWP1* (Fig. 4d, e). However, some of the key genes that were highly upregulated in the CaTLO5 mutant included *OP4* which is associated with the opaque mating phenotype [32], *HGT1* which is involved in glucose uptake [33] and *HYR1* which is a hypha-induced surface protein [34]. Reduced expression of *TLO*α and *TLO*γ genes was also detected, confirming successful *TLO* gene depletion in this mutant (Fig. 4e). GSEA further demonstrated changes in various biological processes, including an increased drug response and decreased oxidative stress responses in the CaTLO5 mutant compared to the isogenic control strain (Fig. 4f). Moreover, genes related to the opaque mating phenotype were upregulated in the CaTLO5 mutant compared to the CapV1200 strain, as well as genes involved in amino acid, sugar and iron transport (Fig. 4f). These changes observed in the CaTLO5 mutant have also been described previously in the ∆*tlo* mutant [15,17]. Decreased expression of ergosterol biosynthetic gene expression was also observed (*ERG1*, *ERG3*, *ERG11* and *ERG251*), and this may be linked to fluconazole tolerance [17].

Comparison of the transcriptomic profile of the CaTLO5 mutant and the CaTLO5+1 reintegrant revealed that key genes involved in ergosterol biosynthesis (*ERG1*) and glycolysis (*PFK2*, *CDC19*) were all upregulated in the CaTLO5 background upon reintroduction of *TLO*α*1* (Fig. 4g). Increased levels of a *TLO*α gene transcript were detected in the CaTLO5+1 reintegrant compared to CaTLO5, suggesting successful expression of *TLO*α*1* in the CaTLO5 background (Fig. 4g). Key genes that were downregulated upon *TLO*α*1* reintegration in the CaTLO5 background included *ECE1*, *UME6*, *WOR1* [35] and *HWP1* (Fig. 4d). GSEA showed that reintegration of *TLO*α*1* led to downregulation of genes involved in various stress responses, including responses to antifungal drugs, copper stress and starvation (Fig. 4h). The latter was indicated by downregulation of amino acid transporter genes, lipid catabolism along with increased expression of *TYE7*-regulated genes [36] and glycolysis in the CaTLO5+1 strain. GSEA also revealed an increase in the expression of genes associated with the white mating phenotype from the opaque phenotype in the CaTLO5 upon reintegration of *TLOα1*. These changes have also been observed previously in the ∆*tlo* mutant after *TLO*α*1* was introduced back into the genome [15].

Overall, the transcriptomic analyses revealed that *TLO* depletion leads to significant transcriptional shifts in genes associated with hyphal morphogenesis, stress responses and metabolic pathways. The differential impact of reintegrating *TLO*α*1* in either the CaTLO2 or CaTLO5 background reinforces the functional differentiation between *TLO* clades and confirms the molecular basis for the phenotypic observations.

### *TLO*α*1* is required for hyphal formation, epithelial invasion and *in vivo* virulence

To investigate the impact of *TLO* gene depletion on host-pathogen interactions, we assessed the behaviour of the *TLO*-depleted strains in the presence of human macrophage and epithelial cell models of infection, as well as in a murine model of OPC. We also aimed to determine if the addition of *TLO*α*1* impacted on virulence, particularly in light of its contrasting roles in CaTLO5 as a suppressor of filamentation and an enhancer of serum-induced hyphal growth [15].

**Fig. 5.**
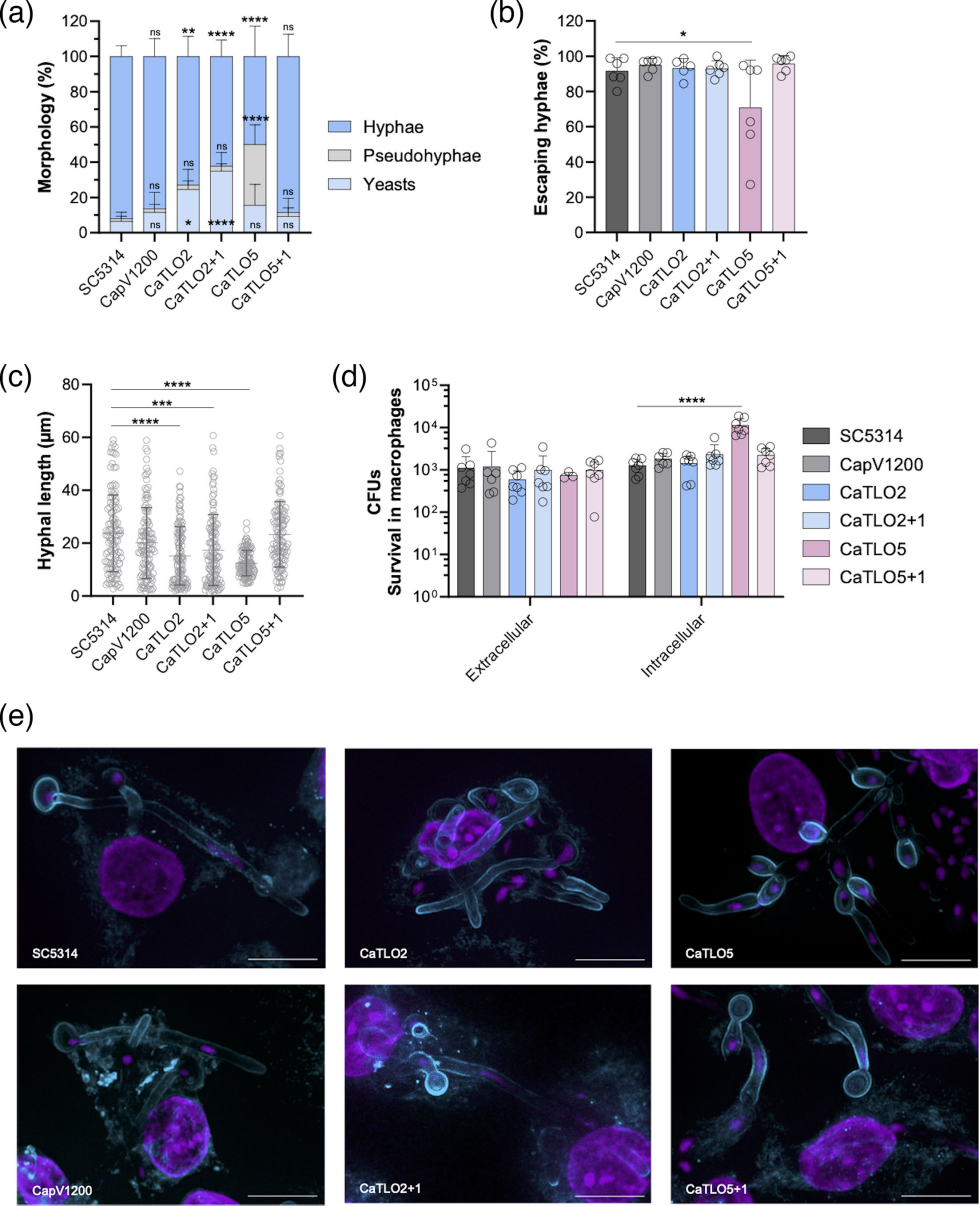
Interaction of the *TLO*-depleted and complemented *C. albicans* mutants with monocyte-derived macrophages. (**a, b**) Hypha formation was determined after infecting monocyte-derived macrophages with an MOI of 5. Infected cells were fixed and stained 3 h post-infection. During microscopic evaluation, 100 fungal cells were counted, and percentage of yeasts/pseudohyphae/hyphae was examined, as well as the amount of invading hyphae (6 donors). (**a**) Statistical significance was calculated using a two-way ANOVA with Tukey‘s multiple comparison test. Asterisks indicate significant differences compared to SC5314. (**b**) Statistically significant differences in number of escaping hyphae relative to SC5314 were calculated using a one-way ANOVA with Dunnett‘s multiple comparison test. Each dot represents one human donor (six donors). (**c**) With the fixed samples, hyphal length was measured using the Leica LAS AF software (Leica Microsystems CMS GmbH) for at least 20 fungal cells per strain and donor. Each dot represents one measured hypha. Statistical significance was calculated using a one-way ANOVA with Dunnett‘s multiple comparison test (six donors). (**d**) Survival in the presence of macrophages was determined by infecting monocyte-derived macrophages with an MOI of 5. After 3 h post-infection, non-phagocytosed fungal cells were washed and collected as supernatant, host cells were lysed, and the intracellularly surviving fungal cells were plated as lysate. Statistical significance was calculated using a two-way ANOVA with Tukey‘s multiple comparisons test. Each dot represents one donor (five donors). (**e**) Representative microscopic images were taken at 3 h post-infection of monocyte-derived macrophages. Fungal cells were stained with AlexaFluor647 conjugated to ConA (blue), and nuclei of the host cells were stained with DAPI (pink). Scale bar, 10 µm. Asterisks indicate significant differences compared to SC5314; *, *P*≤0.05; **, *P*≤0.01; ***, *P*≤0.001; ****, *P*≤0.0001.

When co-cultured with monocyte-derived macrophages at an MOI of 5, parental strains (SC5314 and CapV1200) exhibited robust filamentation, with ~90% of cells forming true hyphae within 3 h (Fig. 5a). In contrast, the CaTLO5 and CaTLO2 mutants displayed impaired filamentation, indicated by significantly reduced abundance of hyphae in these cultures (Fig. 5a). CaTLO5 primarily formed pseudohyphae, while CaTLO2 presented a mixed population of yeasts and short hyphae. Reintegration of *TLO*α*1* restored wild-type-like filamentation in the CaTLO5+1 strain, whereas the CaTLO2+1 strain retained a cell morphology profile similar to CaTLO2 (Fig. 5a, e).

Following incubation, similar levels of CFUs were recovered in supernatants indicating similar levels of phagocytosis. Assessment of the ability of hyphae to escape from monocyte-derived macrophages, measured by AlexaFluor647 staining of externalized filaments, showed that all mutants, apart from CaTLO5, exhibited wild-type levels of escape following internalization (Fig. 5b). Quantification of hyphal length revealed a significant reduction in CaTLO5 filament length, consistent with impaired phagolysosomal escape (Fig. 5c). Reintegration of *TLO*α*1* rescued hyphal length and hyphal escape in the CaTLO5 background (Fig. 5b, c). Strains CaTLO2 and CaTLO2+1 both exhibited reduced hyphal length, but not to the same extent as CaTLO5, and phagolysosomal escape was not impaired in these strains (Fig. 5b, c). Notably, despite its impaired hypha formation, CaTLO5 showed significantly increased survival in macrophage co-cultures compared to wild-type strains (Fig. 5d). This increased intracellular persistence was reversed upon *TLO*α*1* reintegration, suggesting a possible link between pseudohyphal morphology and immune evasion.

**Fig. 6.**
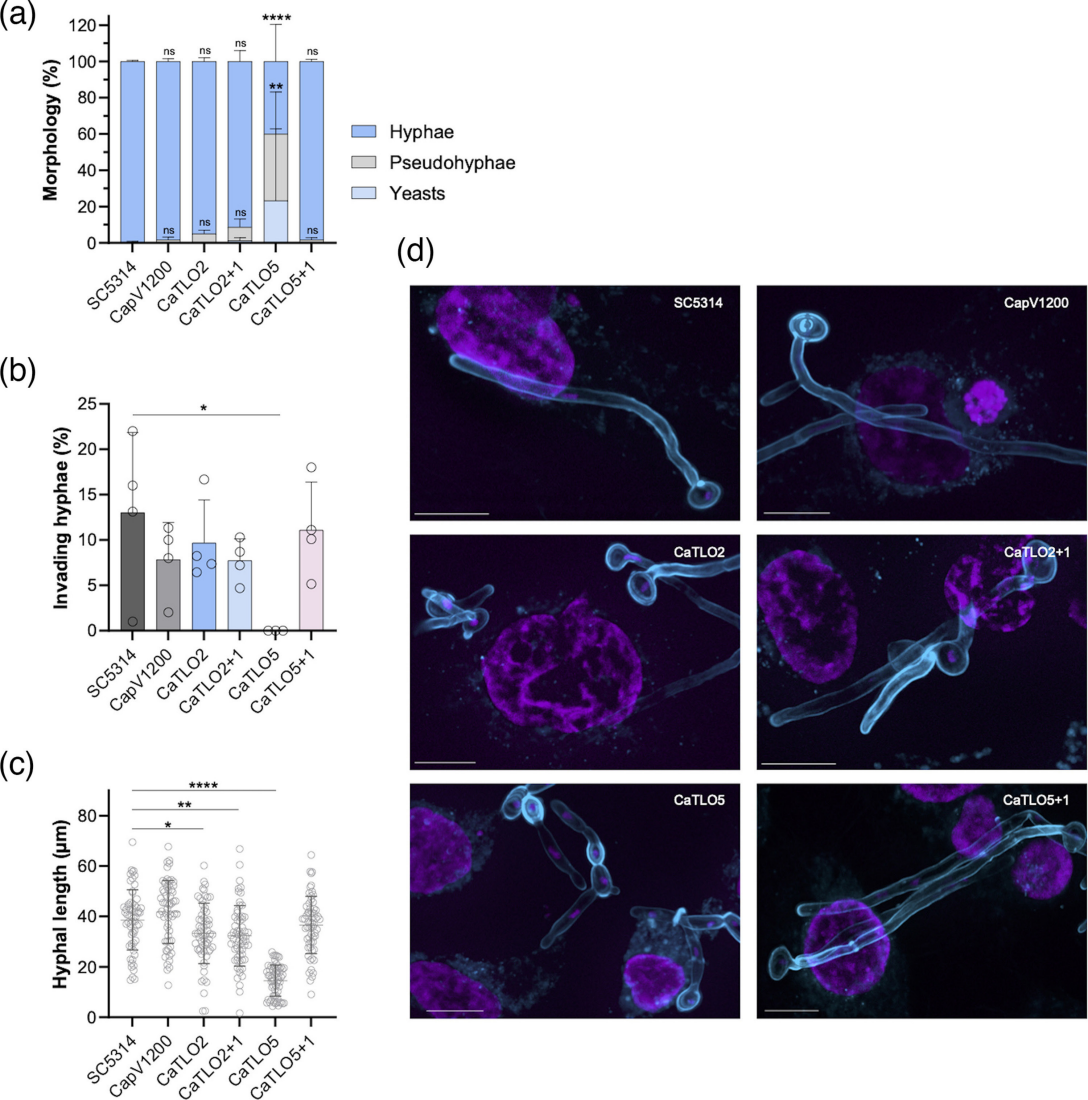
Interaction of the *TLO*-depleted and complemented *C. albicans* mutants with oral epithelial cells. (**a, b**) Hypha formation was determined after infecting oral epithelial cells with an MOI of 1. Infections were fixed and stained 3 h post-infection. During microscopic evaluation, 100 fungal cells were counted, and percentage of yeasts/pseudohyphae/hyphae was examined, as well as the amount of invading hyphae. (**a**) Statistical significance was calculated using a two-way ANOVA with Tukey‘s multiple comparison test. Asterisks indicate significant differences compared to SC5314 (*n*=3). (**b**) Statistical significance was calculated using a one-way ANOVA with Dunnett‘s multiple comparison test. Each dot represents one replicate (*n*=4). (**c**) With the fixed samples, hyphal length was measured using the Leica LAS AF software (Leica Microsystems CMS GmbH) for at least 20 fungal cells per strain and donor. Each dot represents one measured hypha. Statistical significance was calculated using a one-way ANOVA with Dunnett‘s multiple comparison test (*n*=3). (**d**) Representative microscopic images were taken 3 h after infecting TR146 cells. Fungal cells were stained with AlexaFluor647 conjugated to ConA (blue), and nuclei of the host cells were stained with DAPI (pink). Scale bar, 10 µm. Asterisks indicate significant differences compared to SC5314; *, *P*≤0.05; **, *P*≤0.01; ****, *P*≤0.0001.

Infections of oral epithelial cells at an MOI of 1 confirmed that CaTLO5 was severely impaired in hypha formation and epithelial invasion. After 3 h, nearly all (≈90%) SC5314 and CapV1200 cells had transitioned to true hyphae, whereas CaTLO5 remained predominantly in a yeast or pseudohyphal state (Fig. 6a). CaTLO2 and CaTLO2+1 displayed a high proportion of hyphal cells similar to wild-type, but their hyphae were significantly shorter (Fig. 6c). The frequency of invading hyphae was reduced in both strains (Fig. 6b). Reintroduction of *TLO*α*1* restored both filamentation and invasion in CaTLO5+1 but had minimal effect in CaTLO2+1 (Fig. 6a–d).

**Fig. 7.**
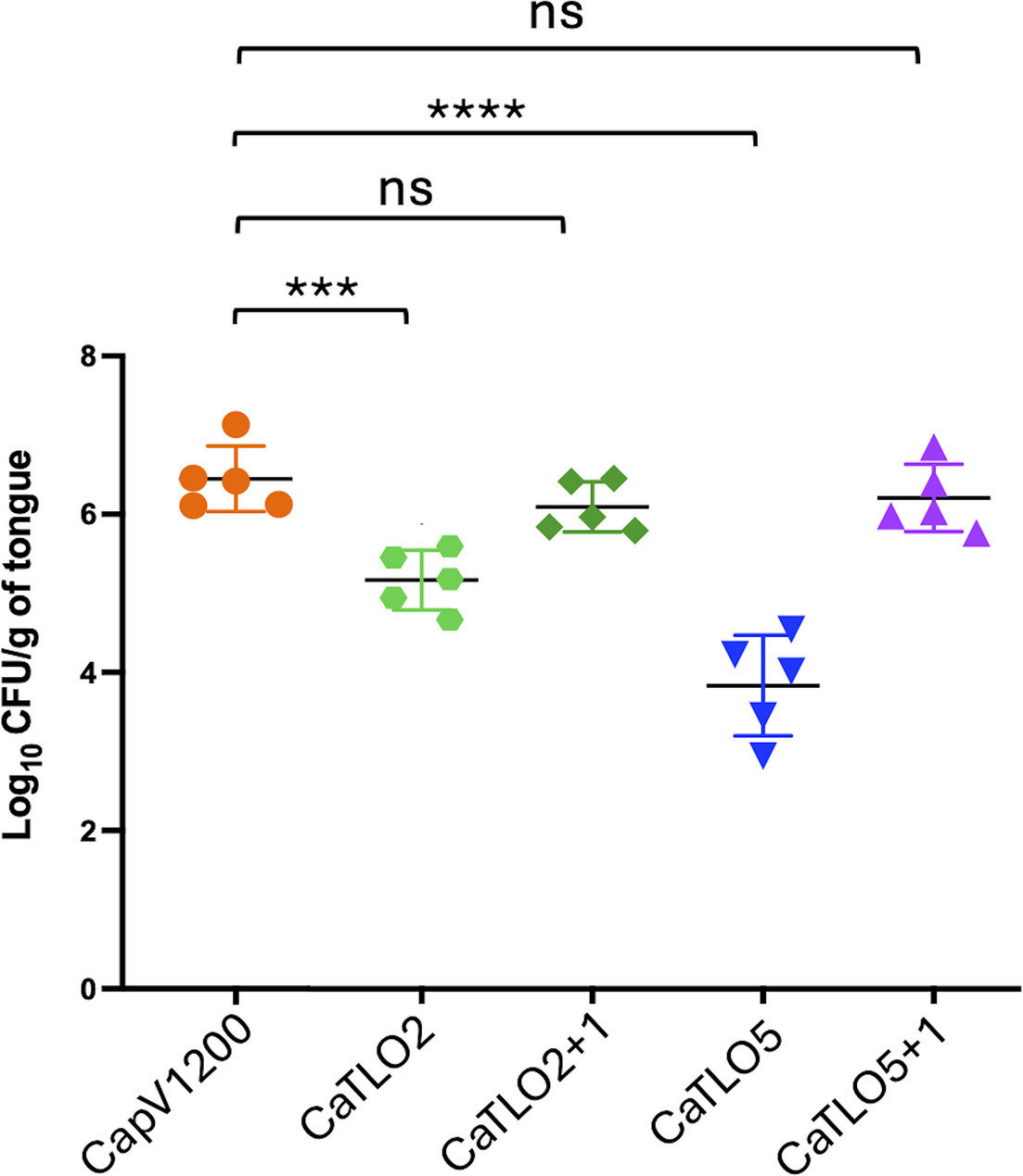
*In vivo* virulence in a murine model of OPC. Data represent fungal burdens (log10 values of c.f.u.) in homogenized tongues of female C57BL/6 mice 5 days post-infection with the indicated *C. albicans* strains. Statistical significance was determined by one-way ANOVA with Tukey’s multiple comparisons test; ***P<0.001, ****P<0.0001; ns, not significant.

To assess the impact of *TLO* depletion on *in vivo* pathogenicity, we used a murine model of OPC (Fig. 7). Following oral inoculation, fungal burden in the tongue tissue was quantified post-mortem. Both CaTLO5 and CaTLO2 mutants exhibited significantly reduced colonization compared to the wild-type, with CaTLO5 showing the most pronounced attenuation (greater than 2 log₁₀ reduction in c.f.u./g) (Fig. 7). Reintegration of *TLO*α*1* restored fungal burden in both mutant backgrounds to wild-type levels, confirming the sufficiency of *TLO*α*1* in driving mucosal infection.

## Discussion

The expansion of the *TLO* gene family in *C. albicans* represents one of the most striking examples of subtelomeric gene expansion in fungal genomes [10,12, 13]. Our previous studies, which generated a Δ*tlo* null mutant, demonstrated that Tlo protein (equivalent to the Med2 subunit of Mediator) was required for normal growth and filamentation *in vitro*. However, these previous studies did not investigate virulence functions or the important question of whether multiple paralogues of *TLO* are required for wild-type virulence. To address this, we used a targeted mutagenesis approach that leaves only a single *TLO* gene intact. We were able to identify gRNA molecules that targeted all *TLO* genes apart from either *TLO*β*2* or *TLO*γ*5,* two paralogues that have previously been shown to be functionally distinct when introduced in a Δ*tlo* null mutant [15]. This approach allowed us to investigate whether *TLO* depletion affected virulence-associated traits in the absence of the broader *TLO* repertoire. Reintegration of *TLO*α*1*, a representative of the α clade, into these minimal backgrounds allowed us to probe the sufficiency of α-*TLO* for wild-type virulence. *TLO*α*1* was selected for complementation as our previous analysis of *in vitro* growth phenotypes showed that *TLO*α*1* could restore normal budding growth and serum-induced hyphal growth in a Δ*tlo* null mutant. The data presented in the current study show that *TLO*α*1* is sufficient to rescue major virulence-related phenotypes in the *TLO*γ*5*-only strain (CaTLO5). CaTLO5 recapitulated the defective morphogenesis, stress sensitivity and reduced virulence previously observed in Δ*tlo* null mutants [15], despite retaining a γ-clade gene. This reinforces previous reports that γ-Tlo proteins, including Tloγ5 and Tloγ11 proteins, are poorly expressed (if at all) and have limited capacity to regulate canonical Med2-dependent transcription [11,15, 19]. Their retention in the genome may reflect non-transcriptional functions, such as mitochondrial functions, telomere-related regulatory roles or the production of regulatory non-coding RNAs [12,37, 38]. However, further experiments directly comparing the CaTLO5 mutant to an isogenic Δ*tlo* null would be required to fully understand the functionality of any remaining Tloγ5 protein. Reintegration of *TLO*α*1* in CaTLO5 restored hypha formation *in vitro*, biofilm structure, oxidative stress resistance and epithelial invasion, indicating that this single α paralogue can reconstitute many of the functions attributed to the *TLO* family as a whole. Other alpha genes may share this capacity; in a recent study, we compared the capacity of *TLO*α*1*, *TLO*α*3* and *TLO*α*34* to restore fluconazole resistance in a Δ*tlo*-Tac1 hyperactive strain. It was noted that all three of the alpha genes could repress filamentation and restore yeast cell growth in the Δ*tlo* null background [39]. These data suggest that the α-*TLO* genes appear to be important repressors of filamentous growth. The phenotypes described here and previously in the Δ*tlo* null are reminiscent of the phenotypes of *NRG1* and *TUP1* mutants, two well-characterized global repressors of filamentous growth. It would be interesting to determine if Mediator containing α-*TLO* interacts with this repressor complex to suppress filamentous growth. Further experiments are underway to systematically delete the *TLO*-α gene group to determine the importance of individual α-gene paralogues and the importance of *TLO*-α gene dosage.

Transcriptomic analysis further indicated the importance of α-*TLO* for wild-type functions. CaTLO5 exhibited upregulation of genes involved in mating (e.g. *WOR1* and *OP4*), iron and amino acid transport (*CFL5*, *GAP2*) and opaque-specific responses, consistent with transcriptomic signatures reported for Δ*tlo* and Δ*med3* strains [15,16]. Reintegration of *TLO*α*1* reversed many of these changes and rebalanced metabolic and stress-related gene expression, consistent with its known association with Mediator and capacity to restore RNAPII recruitment to key promoters [11,19].

The ability of *TLO*α*1* to restore *in vivo* virulence in a murine OPC model further validates its central role. Interestingly, although the impact of *TLO*α*1* integration in CaTLO2 did not significantly impact on phenotype and gene expression *in vitro*, we did observe a noticeable impact on virulence in the murine model. This aligns with prior observations that *TLO*α*1* is highly expressed under pathogenic conditions, including biofilm growth and the *Galleria* infection model [15,16], and suggests that this gene functions as a major regulator of virulence *in vivo*.

In contrast to CaTLO5, the *TLO*β*2*-only strain, CaTLO2, retained more wild-type characteristics, including a smooth colony morphology, but exhibited a significant proportion of hyphal cells in non-inducing conditions. However, CaTLO2 still exhibited defects in growth kinetics in YEPD (likely due to abnormal hyphal growth), defects in hyphal length and *in vivo* virulence – highlighting that *TLO*β*2* is only partially sufficient to support full pathogenic functions in *C. albicans*. These results parallel previous observations in Δ*tlo* reintegrant strains, where complementation with *TLO*β*2* promoted hyphal growth and restored a transcriptional profile similar to wild-type [16].

Interestingly, reintegration of *TLO*α*1* into CaTLO2 (CaTLO2+1) did not significantly alter the transcriptional landscape or fully restore wild-type morphology, growth and stress resistance, despite clear integration and expression of the gene. Phenotypically, the CaTLO2+1 strain resembled the parental CaTLO2 background, suggesting that the leaky expression of the reintegrated *TLO*α*1* gene from the pTET promoter is not sufficient for the ‘wild-type’ α-*TLO* phenotype to manifest. It was only when the expression of *TLO*α*1* in strain CaTLO2+1 was enhanced with doxycycline that a yeast cell morphology closer to wild-type was observed. We do not have sufficient data at this stage to determine whether this is due to a competitive interaction between Tloα1 and Tloβ2 proteins, either at the level of incorporation into the Mediator complex or a downstream competition for coactivator binding. Analysis of Tloα1 and Tloβ2 protein levels in purified Mediator and ChIP analysis of their DNA interactions during co-expression would be required to elucidate such effects. At the transcriptional level, reintegration of *TLO*α*1* in CaTLO2 did not have any significant impact. We have previously shown that differences in the *TLO*α*1* and *TLO*β*2* regulons are subtle, despite each gene promoting divergent cellular morphologies [15]. The current transcriptional profiling experiments were carried out in the absence of doxycycline-regulated *TLO*α*1* induction, which may explain the limited changes in the gene expression detected. Moreover, it raises the possibility that Tlo proteins may be involved in post-transcriptional regulation.

The differential rescue of phenotypes by *TLO*α*1*, *TLO*β*2* and *TLO*γ*5* raises important evolutionary questions: why has *C. albicans* retained such a large and diverse family of *TLO* genes? While gene duplication events often precede subfunctionalization or neofunctionalization [23,24], our data suggest a more nuanced model involving context-dependent regulatory tuning. Rather than acting as redundant copies, *TLO* paralogues may exist as a pool of competing proteins that support different modes of transcriptional regulation depending on environmental conditions, developmental stages or host interactions. Variation in expression at the single-cell level has been described [19]. At a crude level, our study shows that the phenotypic impact of *TLO*α*1* was heavily influenced by the *TLO* background into which it was introduced, indicating that paralogue interplay can modulate functional output. Such dynamic flexibility could confer a selective advantage in the fluctuating environments encountered during *C. albicans* mucosal colonization, biofilm growth or immune evasion.

The role of the γ-clade *TLO* genes in this system may be solely regulatory. Recent data show that *C. albicans* has an active RNAi system that targets the *TLO* gene family, and γ-clade *TLO* transcripts may play a role in regulating α- and β-clade genes [37]. Together with telomeric silencing, these regulatory processes may generate transcriptional heterogeneity in * C. albicans* populations, supporting their plasticity and ability to colonize and infect diverse anatomic sites such as the oral cavity and vagina.

## Conclusions

In summary, this study establishes *TLO*α*1* (used here as a representative of the α-clade of the *TLO* genes) as a master regulator of virulence-associated traits in *C. albicans*. Depletion of the *TLO* gene complement, specifically in strains where the alpha gene family has been deleted, compromises virulence *in vitro* and *in vivo*. These findings extend previous models of functional divergence of the α, β and γ *TLO* gene groups and suggest that evolutionary divergence in these groups has enabled both specialization and fine-tuning of transcriptional regulation. Understanding the cooperative and competitive dynamics within this gene family will be critical for unravelling the transcriptional logic of fungal pathogenesis and for developing strategies to disrupt it.

## Supplementary material

10.1099/mic.0.001654 Uncited Supplementary Material 1.

## Abbreviations

C2BBe1: brush border expressing 1 cell line
CFW: Calcofluor White
CGD: *Candida* Genome Database
ConA: concanavalin A
CR: Congo Red
DMEM: Dulbecco’s modified Eagle's medium
FoG: Fraction of Growth
gRNA: guide RNA
GSEA: Gene Set Enrichment Analysis
hMDMs: human monocyte-derived macrophages
LDH: lactate dehydrogenase
OPC: oropharyngeal candidiasis
YEPD: Yeast Extract Peptone Dextrose
