# Supplementary material for: Depletion of the Candida albicans TLO gene family reveals a requirement for alpha TLO genes for wild-type virulence
Source: Microbiology (Reading). 2026 Jan 16;172(1):001654. doi: 10.1099/mic.0.001654 (PMC13293340; doi:10.1099/mic.0.001654)
Supplement: Uncited Supplementary Material 1. [file mic-172-01654-s001.pdf]

Table S1. Genotypes of *C. albicans* strains used in this study

Strain	Parent	Genotype	Reference
SC5314	-	Wild type	[1]
CapV1200	SC5314	*ENO1/eno1*::pV1200; *TLOα1; TLOβ2; TLOα3; TLOα34; TLOγ4; TLOγ5; TLOγ7; TLOγ8; TLOα9; TLOα10; TLO11; TLOα12; TLOγ13; TLOγ16*	This study
CaTLO2	SC5314	ENO1/eno1::pV1200; ΔΔ*tloα1;* ***TLOβ2***; ΔΔ*tloα3;* ΔΔ*tloα34;* ΔΔ*tloγ4;* ΔΔ*tloγ5;* ΔΔ*tloγ7;* ΔΔ*tloγ8;* ΔΔ*tloα9;* ΔΔ*tloα10;* ΔΔ*tlγo11;* ΔΔ*tloα12;* ΔΔ*tloγ13;* ΔΔ*tloγ16*	This study
CaTLO5	SC5314	*ENO1/eno1*::pV1200; ΔΔ*tloα1;* ΔΔ*tloβ2;* ΔΔ*tloα3;* ΔΔ*tloα34;* ΔΔ*tloγ4;* ***TLOγ5***; ΔΔ*tloγ7;* ΔΔ*tloγ8;* ΔΔ*tloα9;* ΔΔ*tloα10;* ΔΔ*tlγo11;* ΔΔ*tloα12;* ΔΔ*tloγ13;* ΔΔ*tloγ16*	This study
CaTLO2+1	ScTLO2	*ENO1/eno1*::pV1200; ΔΔ*tloα1;* ***TLOβ2***; ΔΔ*tloα3;* ΔΔ*tloα34;* ΔΔ*tloγ4;* ΔΔ*tloγ5;* ΔΔ*tloγ7;* ΔΔ*tloγ8;* ΔΔ*tloα9;* ΔΔ*tloα10;* ΔΔ*tlγo11;* ΔΔ*tloα12;* ΔΔ*tloγ13;* ΔΔ*tloγ16; ADH1/adh1*::pNIM-*TLOα1*	This study
CaTLO5+1	SCTLO5	*ENO1/eno1*::pV1200; ΔΔ*tloα1;* ΔΔ*tloβ2;* ΔΔ*tloα3;* ΔΔ*tloα34;* ΔΔ*tloγ4;* ***TLOγ5***; ΔΔ*tloγ7;* ΔΔ*tloγ8;* ΔΔ*tloα9;* ΔΔ*tloα10;* ΔΔ*tlγo11;* ΔΔ*tloα12;* ΔΔ*tloγ13;* ΔΔ*tloγ16; ADH1/adh1*:: pNIM-*TLOα1*	This study
CC16 (*tlo*Δ)	MAY1244	*LEU2*/Δ*leu2*; ΔΔ*tloα1;* ΔΔ*tloβ2;* ΔΔ*tloα3;* ΔΔ*tloα34;* ΔΔ*tloγ4;* ΔΔ*tloγ5;* ΔΔ*tloγ7;* ΔΔ*tloγ8;* ΔΔ*tloα9;* ΔΔ*tloα10;* ΔΔ*tlγo11;* ΔΔ*tloα12;* ΔΔ*tloγ13;* ΔΔ*tloγ16*	[2]

1. **Gillum AM, Tsay EY, Kirsch DR**. Isolation of the Candida albicans gene for orotidine-5'-phosphate decarboxylase by complementation of S. cerevisiae ura3 and E. coli pyrF mutations. *Mol Gen Genet : MGG* 1984;198:179–82.

2. **Fletcher J, O'Connor-Moneley J, Frawley D, Flanagan PR, Alaalm L, *et al.*** Deletion of the Candida albicans TLO gene family using CRISPR-Cas9 mutagenesis allows characterisation of functional differences in α-, β- and γ- TLO gene function. *PLOS Genet* 2023;19:e1011082.

Fig. S1

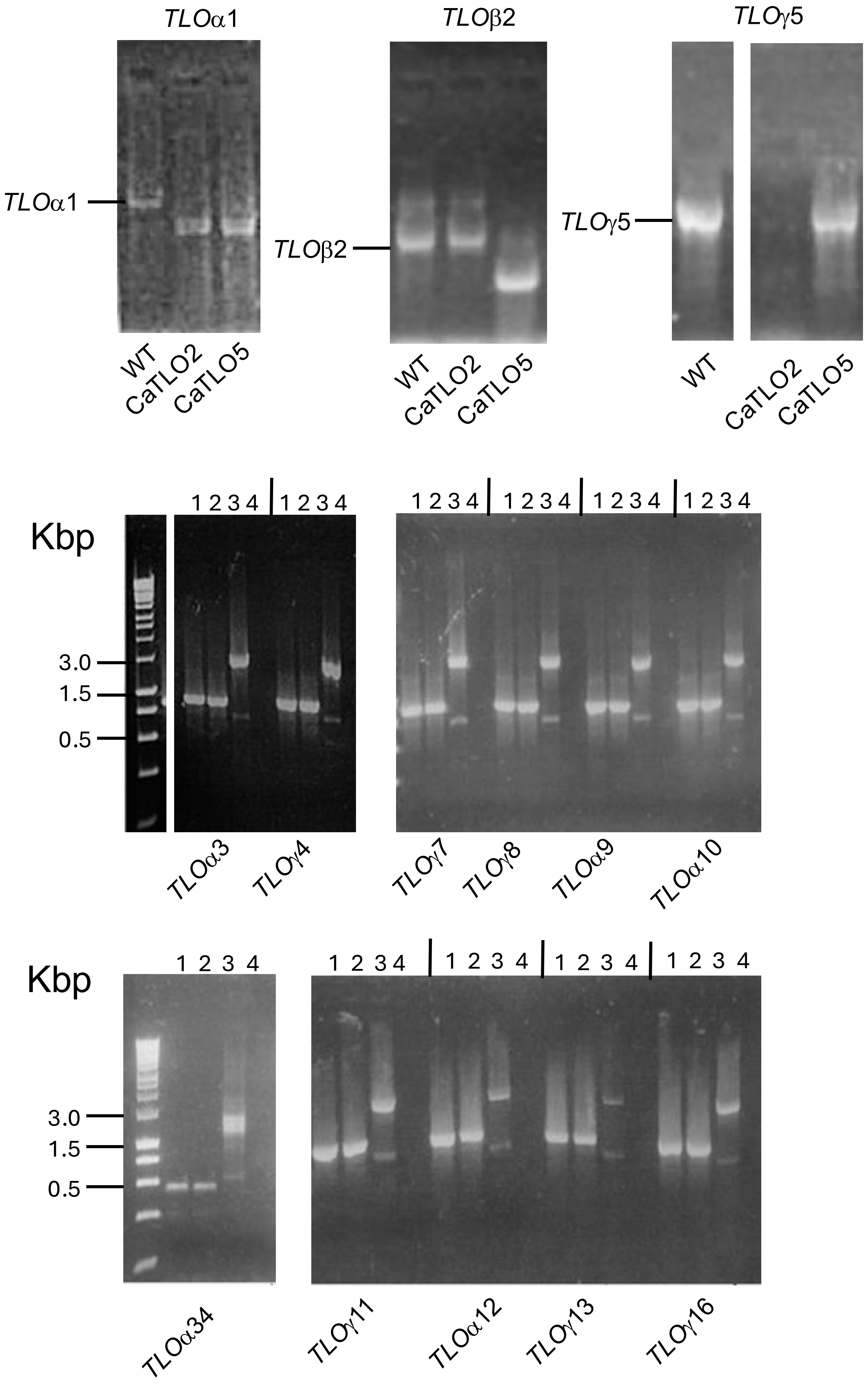

Figure S1. PCR analysis of specific TLO loci to detect the presence of CRISPR-Cas9 deletions. A pan-TLO primer was used in conjunction with chromosome arm specific primers (Fletcher et al., 2023) to detect the presence of *TLO* genes. The deletion of *TLOα1* in CaTLO2 and CaTLO5 is shown in the top left panel, the deletion of *TLO*β2 *in* ScTLO5 is shown in the top center panel and the deletion of *TLOγ5* in CaTLO2 is shown in the top right panel. Middle and lower panels show *TLO* amplicons for the remaining genes in strains CaTLO2 (1), CaTLO5 (2), SC5314 (3) and negative control reactions (4). Primers were described by Fletcher et al. (2023).

Fig. S2

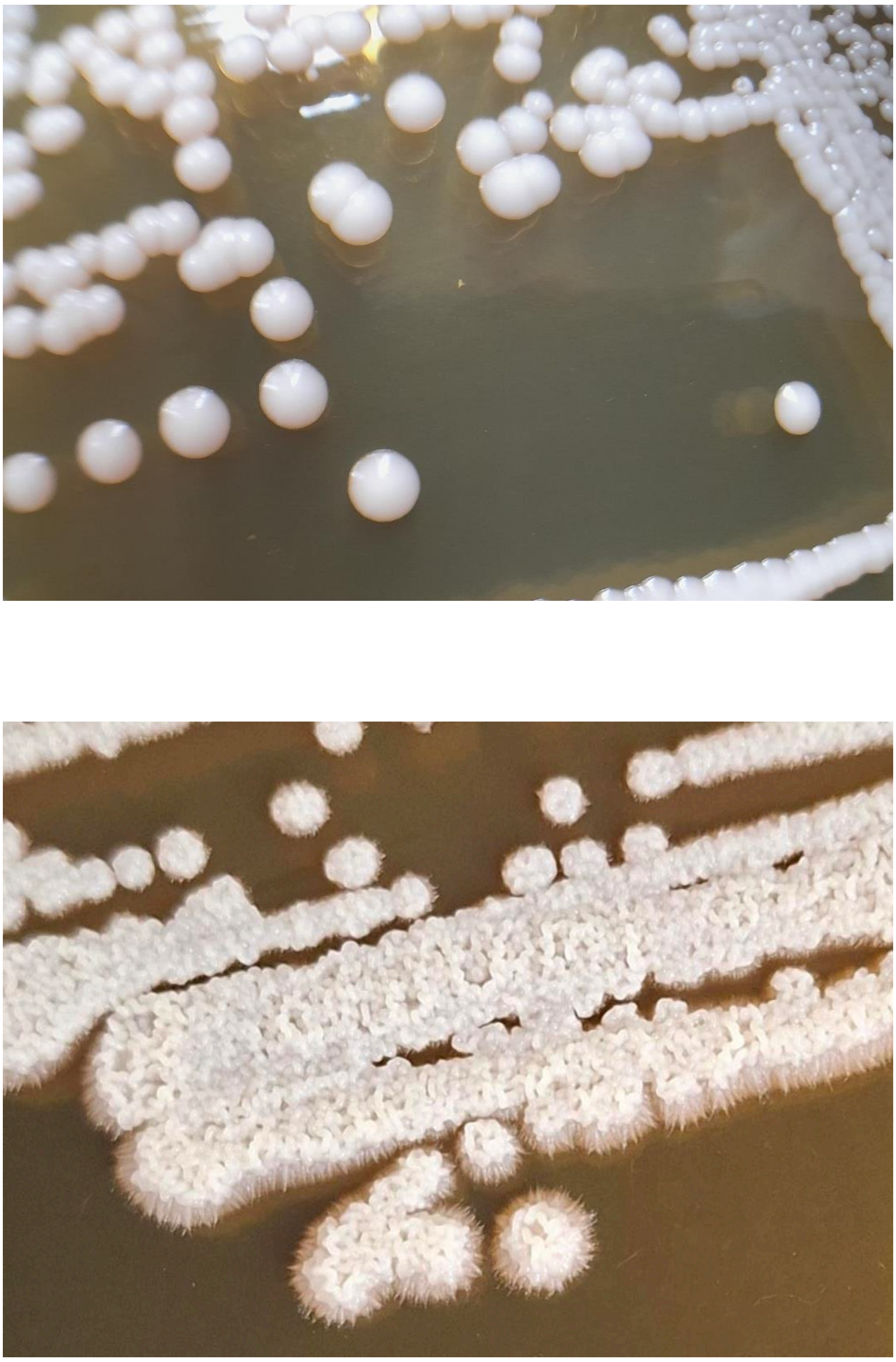

Figure S2. Colony morphology of CaTLO2 and CaTLO5 grown on YPD medium for 48h at 30˚C

Fig. S3

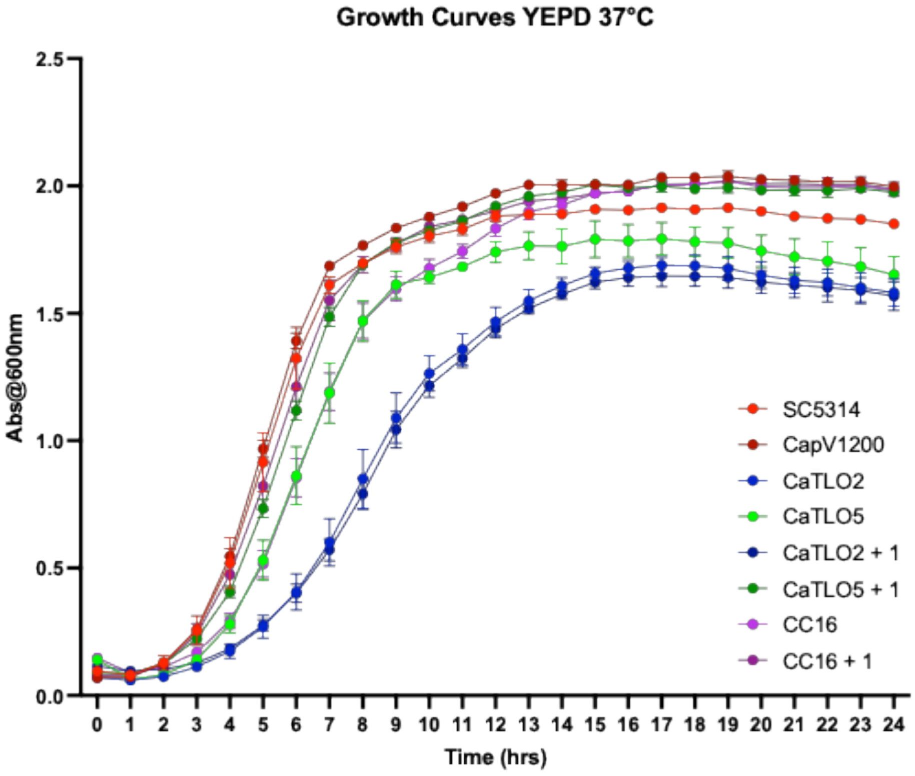

Figure S3. Growth kinetics and doubling times of *TLO*-depleted *Candida albicans* mutants and complemented strains. (A) Representative sigmoidal growth curves of laboratory strains (SC5314 and pV1200), *TLO*-depleted mutants (ScTLO2, ScTLO5, *Δtlo*), and corresponding *TLOα1* reintegrants (ScTLO2+1, ScTLO5+1, *Δtlo*+1). Strains were cultured in YEPD medium at 37°C with shaking, and optical density (OD600) was measured at hourly intervals over 24 hours. Curves represent the mean of three independent biological replicates.

Fig. S4

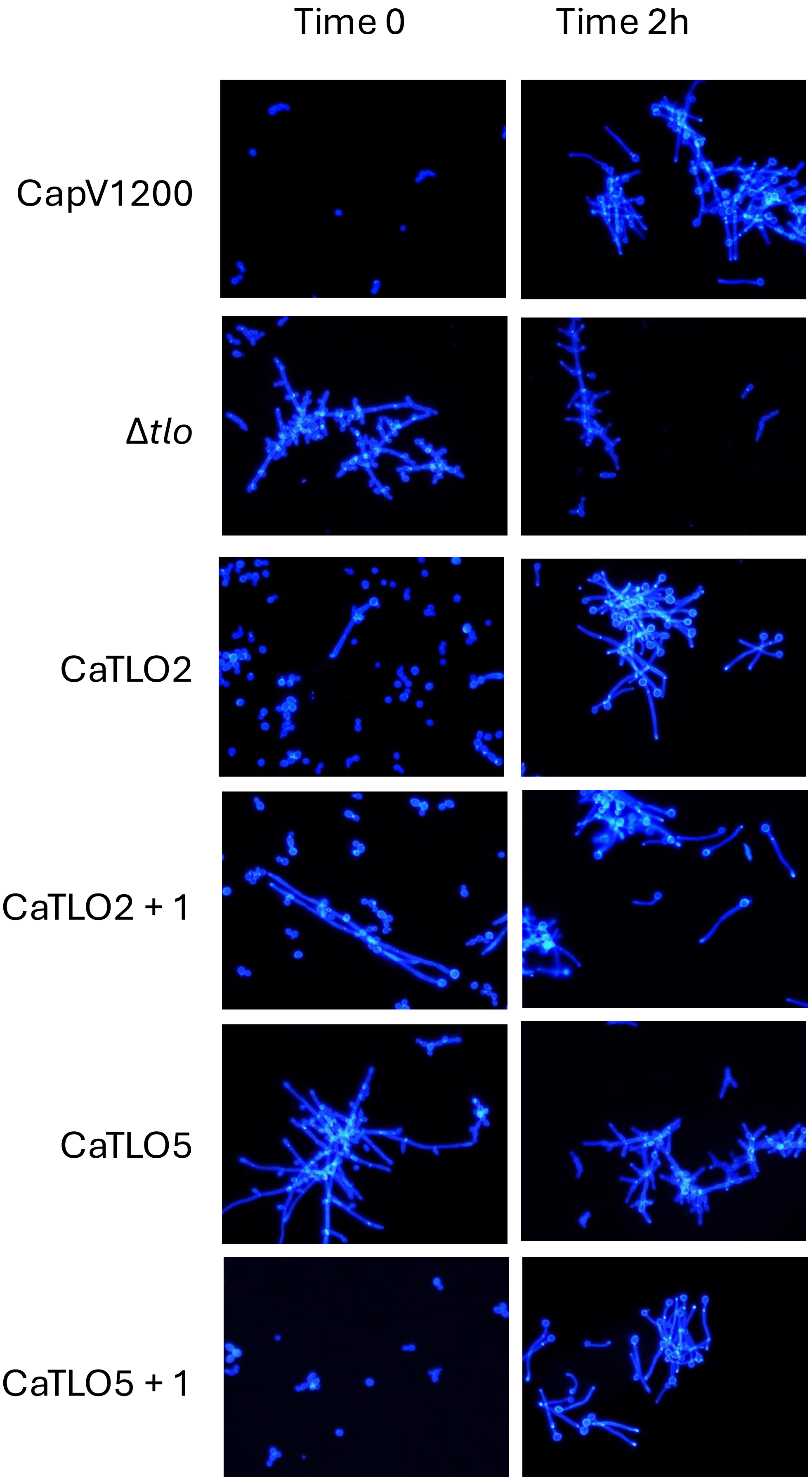

Figure S4. Serum-induced hyphal formation in *TLO*-depleted *Candida albicans* mutants and *TLOα1* reintegrants.

Representative micrographs of strains and mutants stained with calcofluor white following hyphal induction in liquid YEPD medium supplemented with 10% fetal bovine serum (FBS) at 37°C for 30 minutes and 2 hours. Laboratory strains (SC5314 and pV1200) and the CaTLO2 mutant formed true hyphae under these conditions, while CaTLO5 and *Δtlo* mutants displayed pseudohyphal morphology despite serum induction. Reintegration of *TLOα1* into the CaTLO5 and *Δtlo* backgrounds restored true hyphal formation.

Fig. S5

A

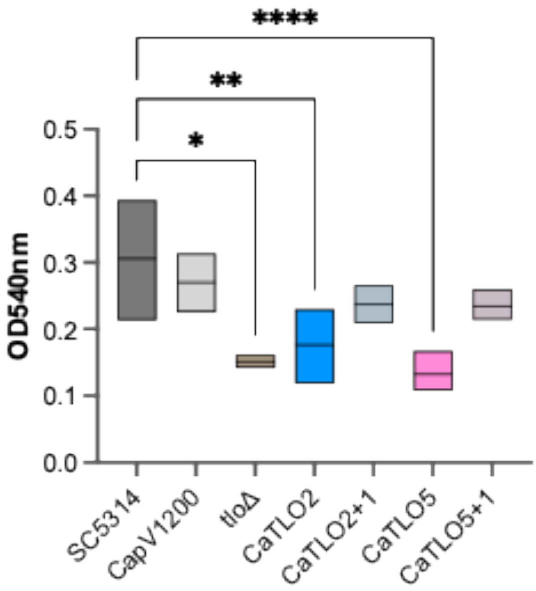

B

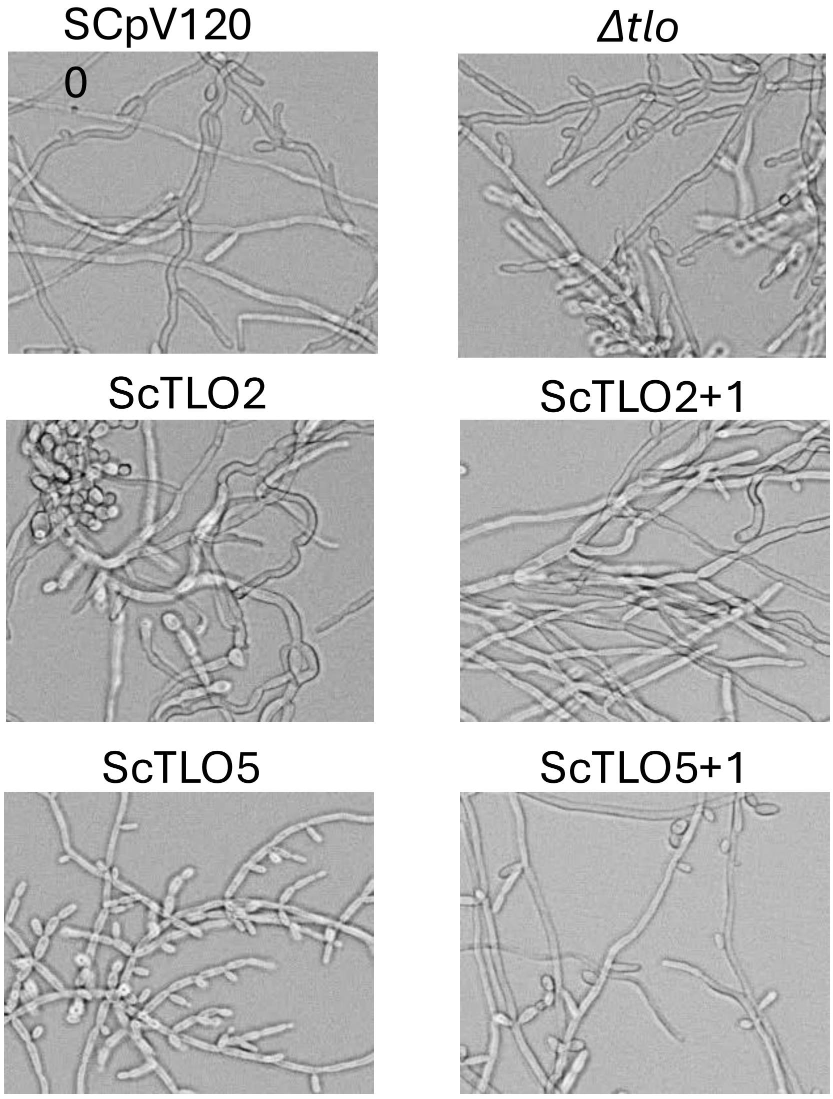

Figure S5. Biofilm formation and structural morphology of *TLO*-depleted and complemented *Candida albicans* strains.

(A) Quantification of biofilm biomass formed by *TLO*-depleted mutants (CaTLO2, CaTLO5, *Δtlo*), and corresponding *TLOα1* reintegrants (CaTLO2+1, CaTLO5+1). Biofilms were grown statically in Spider medium at 37°C for 48 hours, and biomass was measured by crystal violet staining followed by solubilization and absorbance measurement at 540 nm. Data represent mean ± SEM of three independent biological replicates. Statistical significance was determined by one-way ANOVA with Dunn's multiple comparisons test. ****$p < 0.0001$; **$p < 0.01$, *$p < 0.05$. (B) Representative phase contrast micrographs of mature 48-hour biofilms.

Fig. S6

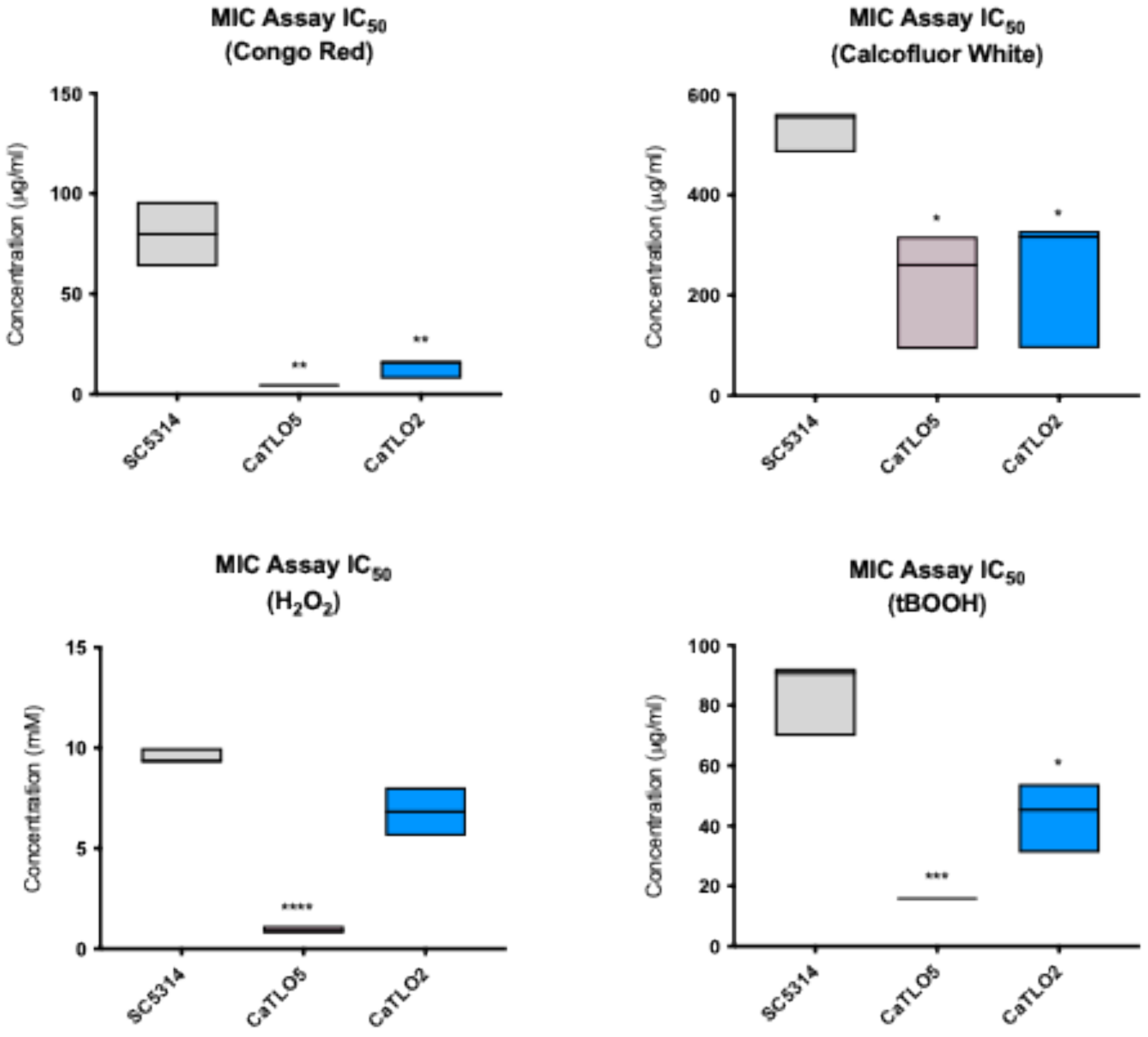

Figure S6. Minimum inhibitory concentrations of Congo Red, Calcofluor white, H_2O_2 and tBOOH. MICs were determined in liquid Yeast Nitrogen Base medium containing 2% glucose (YNB-G) in 96-well plates. To prepare the assay, yeast cells grown overnight in liquid YEPD at 37˚C were washed in sterile water and a suspension was prepared in YNB-G at OD600 0.1. A 10 µl volume of the suspension was added in duplicate rows of 96-well flat bottom plates containing serial 2-fold dilutions of each of the agents in the ranges shown. A drug free control was included. Plates were incubated overnight at 37˚C and OD600 values were determined in a FLUOstar Omega Microplate reader. The MIC concentration was determined as the lowest concentration to reduce growth below 80% of the control. Data shown are the result of three independent experiments.

Statistical significance was determined by one-way ANOVA with Dunn's multiple comparisons test. ****$p < 0.0001$; ***$p < 0.001$;**$p < 0.01$, *$p < 0.05$.

Fig. S7

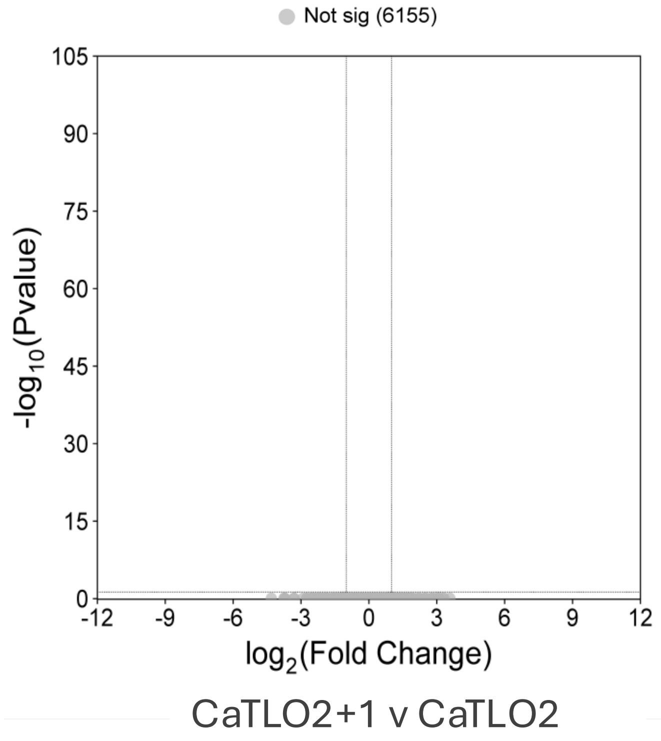

Figure S7. Transcriptomic analysis of the *C. albicans TLO* depleted mutant CaTLO2 versus the *TLOα1* complemented derivative CaTLO2+1. All genes are in grey as they are above the significance cut off (FDR >0.1).
